# The Value of the Endoscope-Holding Arm in Transoral Pharyngeal Surgery

**DOI:** 10.3390/jcm13020507

**Published:** 2024-01-16

**Authors:** Aris I. Giotakis, Evangelos I. Giotakis, Efthymios Kyrodimos

**Affiliations:** First Department of Otorhinolaryngology, Hippocrateion General Hospital, 115 27 Athens, Greece; giotakis@gmail.com (E.I.G.); timkirodimos@hotmail.com (E.K.)

**Keywords:** endoscopes, oropharynx, hypopharynx, minimally invasive surgical procedures, head and neck neoplasms

## Abstract

Background: Transoral pharyngeal surgery is mainly feasible with the use of a microscope or robotic systems. Data about alternative methods, with lower costs and easier availability, are sparse. We intended to examine to what extent the endoscope-holding arm is a suitable alternative to the microscope or robotic systems. Material and Methods: We retrospectively reviewed subjects who underwent pharyngeal tumor resection with the endoscope-holding arm in our university department. Results: We identified 13 subjects who underwent transoral pharyngeal surgery between November 2020 and November 2023. Most subjects presented with an oropharyngeal tumor (6/11 in the lateral wall or tonsil; 4/11 in the tongue base). The oropharyngeal lateral wall or tonsillar tumors were exposed with a standard mouth gag. The tongue-base tumors or hypopharyngeal tumors were exposed with an operating laryngoscope. Advantages over the microscope included an angled view. Advantages over robotic systems included haptic feedback and a faster setup. Advantages over both the microscope and robotic systems included lower costs and easier availability. Visualization with the endoscope was sufficient and similar to that of the microscope. Bimanual action was possible with surgical forceps and a monopolar electrode. Conclusions: Transoral pharyngeal surgery was feasible with the endoscope-holding arm. The endoscope-holding arm could be a cost-efficient alternative to the microscope or robotic systems.

## 1. Introduction

Surgical management of oropharyngeal, laryngeal and hypopharyngeal tumors is considered challenging due to difficult exposure. Surgical approaches to the oropharynx, larynx and hypopharynx are mainly transoral [1,2,3]; transcervical, via lateral pharyngotomy [4,5,6]; and transmandibular, via midline labiomandibuloglossotomy or composite resection with segmental mandibulectomy [7]. Due to the functional morbidity and complications associated with open approaches, transoral pharyngeal surgery is preferred as the less invasive approach.

Traditionally, the microscope has been used in oncological transoral pharyngeal surgery. However, the microscope has certain disadvantages: high costs result in limited availability around the world; its large size makes it unsuitable for small operating theatres; auxiliary staff with expertise might be needed; and the surgeon must adapt to indirect vision with a narrow field [8,9].

Advancements in technology have led to robotic [10,11,12,13,14] or endoscopic [15,16,17,18] transoral surgery. The main disadvantages of transoral robotic surgery are the substantial cost of acquiring and maintaining robotic systems, the lack of haptic feedback for the operating surgeon [19], the space needed in the surgical room and the surgical training required. On the contrary, the endoscope has several advantages. It is user friendly, cheap, easy to store, requires no specific surgical training and is widely available.

Nevertheless, the endoscope has a serious weakness. It occupies one hand of the surgeon, resulting in poor surgical mobility. Rhinologists are often confronted with this problem. This has led to the integration of a co-surgeon who guides the endoscope in certain situations via the four-hand technique. However, this might lead to shaky images due to fatigue, and there can be a blurred field of vision due to smudges of blood on the endoscope lens [20]. Furthermore, an additional co-surgeon with experience is not always available.

To address these issues, various attempts have been made to bring in the use of endoscope-holding arms in skull-base surgery [21,22]. As expected, these also have several disadvantages, which include, among others, the bulky design of the arm and inflexibility through limited degrees of freedom compared to free-hand movement [21,22]. Recently, Hintschich and coauthors reported the endoscope-holding arm named ENDOFIX (AKTORmed, Barbing, Germany), for use in sinus and laryngopharynx surgery, which may overcome these difficulties. The authors concluded that the ENDOFIX unites maneuverability, flexibility and stability. Thus, the surgeon can operate in a bimanual action—even in the absence of an assistant [23].

Considering the limited data on endoscope-holding arms in transoral pharyngeal surgery, we retrospectively reviewed and documented the applications, advantages and disadvantages of the ENDOFIX in oropharyngeal, laryngeal and hypopharyngeal surgeries in a single tertiary center. We intended to investigate to what extent the ENDOFIX could offer an efficient and cost-effective alternative to the microscope and robotic systems commonly used in transoral pharyngeal surgery.

## 2. Materials and Methods

### 2.1. Study Design

We retrospectively reviewed all patients who underwent oncological transoral pharyngeal and laryngeal surgery with the ENDOFIX at the First Department of Otorhinolaryngology, Head and Neck Surgery, Hippocrateion General Hospital, Athens, Greece. The policy in our department favors the use of a microscope for transoral pharyngeal and laryngeal surgery. More technologically advanced methods, like the ENDOFIX, have been used occasionally, when possible. This study was conducted in accordance with the Declaration of Helsinki and approved by the Institutional Review Board of Hippocrateion General Hospital in Athens, Greece (protocol code: 54; date of approval: 21 December 2023). The procedures followed were in accordance with the ethical standards of the committee on human experimentation of the institution or in accordance with the Helsinki Declaration of 1975, as revised in 1983.

### 2.2. Data Collection

Surgeons were encouraged to prospectively document and collect data on every patient with a head and neck tumor. These data included age, gender, smoking and alcohol consumption, comorbidities, TNM stage according to the 8th “Union for International Cancer Control” Edition, status of T (tumor size and site), of N (regional lymph node involvement) and of M (distant metastasis), grade and histology. Further data included the presence of complications, functional outcomes (e.g., dysphagia), disease-free survival (DFS) status, overall survival (OS) status, the dates of the events, the region of any metastatic disease and the cause of death.

### 2.3. Arrangement of the Operating Room

The patient was placed on the operating table in a supine position. For exposure of the posterior and lateral oropharyngeal walls, we used a standard-model mouth gag. For exposure of the tongue base, larynx and hypopharynx, we used an operating laryngoscope. For visualization, we used either a 50 cm long rigid 0° endoscope with a diameter of 5.5 mm (catalog number: 10320 AA; Karl Storz, Tuttlingen, Germany) or a 24 cm long rigid 30° endoscope with a diameter of 5.0 mm (catalog number: tk 700-058; Tekno-Medical Optik-Chirurgie GmbH; Sattlerstraße 11, 78532, Tuttlingen, Germany).

**Figure 1:**
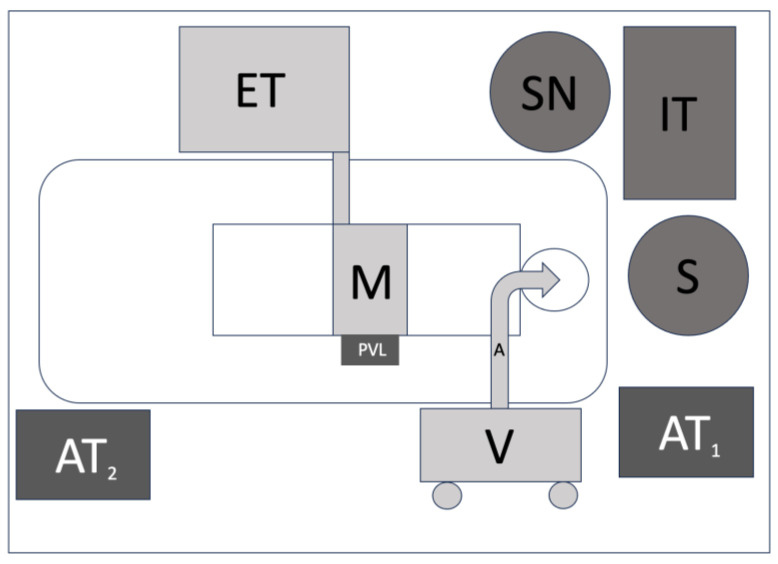
A schematic arrangement of the operating room. S: surgeon; SN: scrub nurse; IT: instrument table; A: ENDOFIX arm on a wheeled vehicle (V); AT_1_: anesthesiology tower; AT_2_: an alternative placement of the anesthesiology tower; PVL: peripheral venous line; ET: endoscopic tower; M: monitor.

The surgeon was positioned behind the head of the patient. The scrub nurse and instrument table were placed on the right side of the surgeon. The ENDOFIX was placed on the left side of the operating table on a wheeled stand. This facilitated the mobility of the ENDOFIX compared to the attachment of the ENDOFIX onto the left standardized side rails of the operating table. The endoscopic tower was placed next to the patient’s right arm, with the monitor extending to the front of the surgeon, above the patient’s abdomen. The anesthesiology tower was placed on the left side of the surgeon, but it could also be placed next to the patient’s left foot with an intubation tube extension cable (Figure 1).

## 3. Results

**Figure 2:**
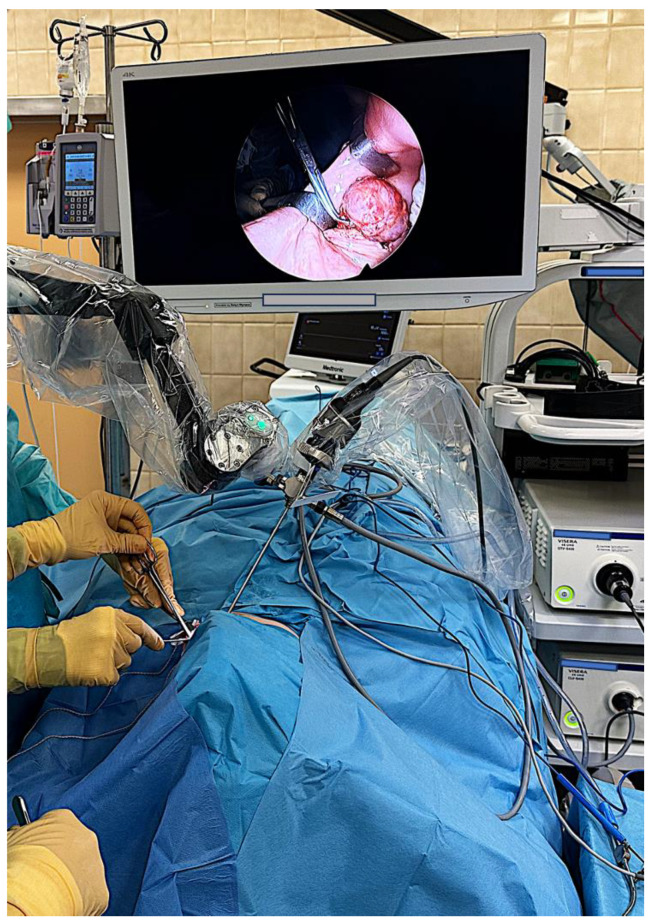
The operating field during transoral resection of a left buccal pleomorphic adenoma in a 55-year-old woman (not a study subject). Note the monitor above the patient’s abdomen, the instrument table on the right of the operating table (in front of the tower) and the ENDOFIX above the patient’s head, stabilized on a wheeled vehicle (not visible). Due to the tumor’s site, an assistant was part of the operating team.

Between November 2020 and November 2023, 122 patients underwent transoral pharyngeal surgery with a microscope and a CO_2_ laser. We identified 13 subjects who underwent pharyngeal surgery with the ENDOFIX (Table 1). The first subject underwent surgery in November 2020. Mean age ± standard deviation was 60 ± 10 years. For all 13 subjects, the treatment strategy was approved by a multidisciplinary tumor board. After transoral intubation with a 6-size tube and insertion of a standard-model mouth gag or the largest possible operating laryngoscope, the ENDOFIX was set (Figure 2).

Bimanual action allowed tumor traction with surgical forceps and resection with surgical scissors and/or a specific monopolar electrode. This was a 45° angled-down monopolar microdissection electrode with a tip and a total length of 229 cm (catalog number: 364472; Sutter Medizintechnik GmbH, Alfred-Walz-Str. 22, 79312, Emmendingen, Germany).

Occasionally, throughout the surgery, it was necessary for the surgeon to change the placement and angulation of the ENDOFIX. All tumors were resected with clear margins (>5 mm). Operations were carried out by two surgeons (EIG and EK). In the following sections, we present six representative cases in more detail.

### 3.1. Case 1

**Figure 3:**
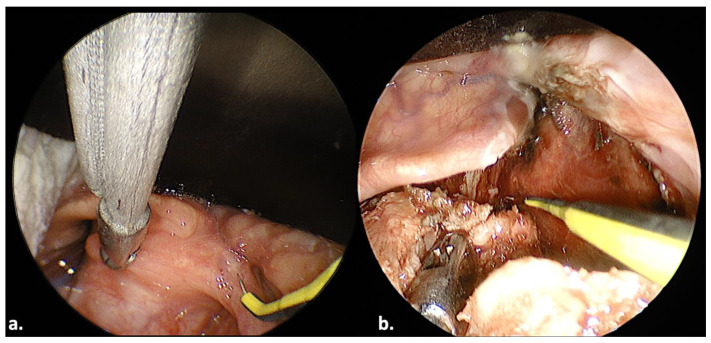
Transoral resection of a laryngeal tumor in a 65-year-old man. Note the bimanual action.

A 65-year-old man presented with dysphagia in May 2023. A laryngopharyngeal endoscopy revealed a laryngeal tumor originating from the right arytenoid cartilage. The tumor protruded on the posterior hypopharyngeal wall. The patient reported no dyspnea. A computed tomography scan of the neck and thorax with contrast medium revealed no regional lymph node disease and no distant metastasis. A biopsy revealed a liposarcoma. The patient underwent transoral endoscopic surgery in June 2023. In this case, we used a Weerda laryngoscope. Surgery was challenging due to limited space caused by the ENDOFIX inside the operating laryngoscope. Insertion into and handling of surgical tools through the operating laryngoscope required patience. Despite this, surgery was feasible. The tumor was resected from the adjacent normal laryngeal structures (Figure 3). The tumor was pulled posteriorly away from the epiglottis with surgical forceps (note the epiglottis, which is located anterior to the surgical forceps: Figure 3a). The bimanual action, with the posterior traction of the tumor and cutting with the monopolar electrode, facilitated the resection of the tumor (Figure 3b). The operation lasted 114 min. A nasogastric tube was required for 10 days. Total oral diet was fully restored. No postoperative complications were noted. The patient was tumor-free 6 months postoperatively.

### 3.2. Case 4

**Figure 4:**
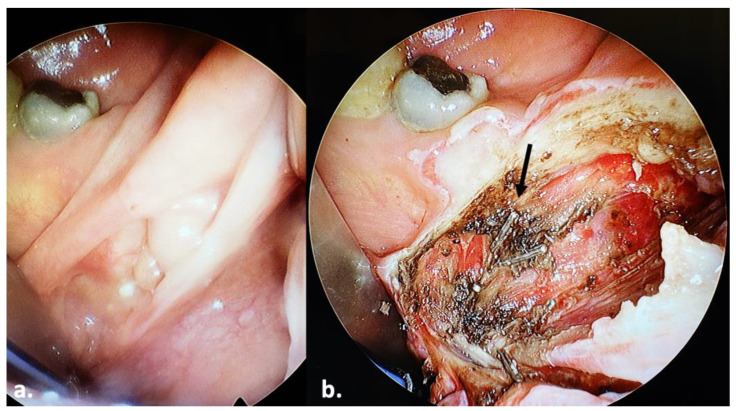
Transoral resection of a right oropharyngeal (tonsillar) tumor in a 51-year-old man. Black arrow: surgical clips.

A 51-year-old man presented with dysphagia in May 2023. A clinical examination revealed a tumor of the right tonsil. A computed tomography scan of the neck and thorax with contrast medium revealed no regional lymph node disease and no distant metastasis. A biopsy revealed a squamous-cell carcinoma, confirming the diagnosis of an oropharyngeal squamous-cell carcinoma of the right tonsil. The patient underwent transoral pharyngeal endoscopic surgery in June 2023. The operating team invested sufficient time to ensure the correct placement and angulation of the ENDOFIX, in order for the surgeon’s hands to be unobstructed during movement. The tumor was adequately exposed and visualized (Figure 4a). Tumor resection was feasible with a pair of instruments, while the endoscope allowed for the visualization of branches of the lingual or ascending pharyngeal arteries. We applied surgical clips (Figure 4b; black arrow) on these branches for hemostasis. The operation lasted 45 min. We noted no postoperative bleeding. No other postoperative complications were noted.

### 3.3. Case 7

**Figure 5:**
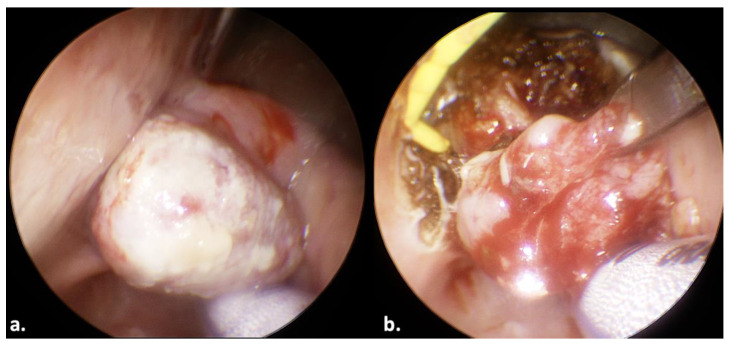
Transoral resection of an oropharyngeal tumor of the left tongue base in a 65-year-old woman. Note the visualization of the glossoepiglottic sulcus.

A 65-year-old woman presented with dysphagia in September 2023. A pharyngeal endoscopy revealed an oropharyngeal tumor of the left tongue base. A biopsy revealed a squamous-cell carcinoma. A computed tomography scan of the neck and thorax with contrast medium revealed no regional lymph node disease and no distant metastasis. The patient underwent transoral pharyngeal endoscopic surgery in October 2023. The endoscope facilitated visualization of both the glossoepiglottic sulcus and the tumor, which was also adequately exposed (Figure 5a). Once again, correct placement of the ENDOFIX and surgical instruments through the operating laryngoscope was crucial. The bimanual action allowed for adequate traction and resection of the tumor with a monopolar electrode (Figure 5b). The operation lasted 30 min. We noted no bleeding. No further postoperative complications were noted.

### 3.4. Case 8

**Figure 6:**
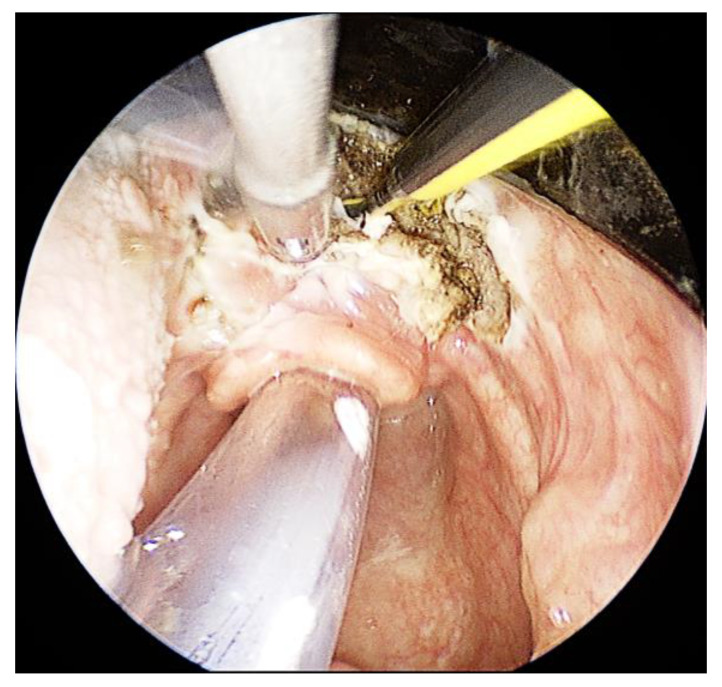
Resection of the tongue base along with the pharyngoepiglottic fold in a 72-year-old woman with cancer of unknown primary. Note the adequate exposure and bimanual action with the surgical forceps and the monopolar electrode.

A 72-year-old woman presented with swollen neck lymph nodes in August 2023. The suspicion of cancer of unknown primary was confirmed with a needle core biopsy from a lymph node. A fluorodeoxyglucose positron-emission tomography scan indicated a higher signal in the tongue base. The patient underwent transoral pharyngeal endoscopic surgery in September 2023. The endoscope facilitated visualization of the tongue base. Due to limited space through the operating laryngoscope, readjustments of the ENDOFIX were necessary throughout the surgery (Figure 6). Bimanual action allowed resection of the tongue base along with the pharyngoepiglottic fold. The surgery lasted 35 min. We noted no bleeding. No further postoperative complications were noted. A histological examination revealed normal mucosa.

### 3.5. Case 12

**Figure 7:**
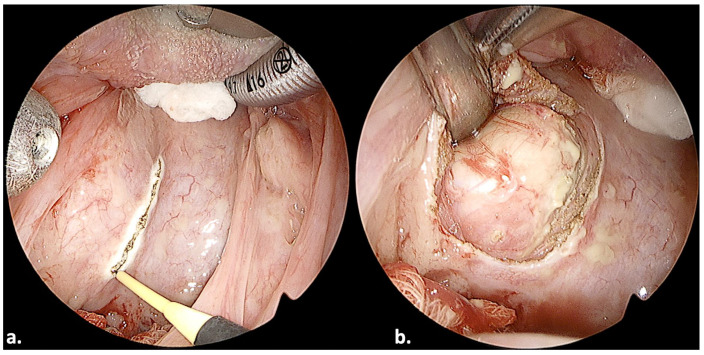
Transoral resection of a left posterior retropharyngeal mass in a 65-year-old woman. Note the synchronous use of two instruments: Blakesley forceps and a monopolar electrode.

A 65-year-old woman presented with dysphagia and foreign-body sensation in the pharynx in September 2023. A magnetic resonance tomography scan with gadolinium revealed a left cystic retropharyngeal cystic mass without any pathological lymph nodes. The patient underwent transoral pharyngeal endoscopic surgery in October 2023. After visualization of the tumor, the mucosa of the posterior pharyngeal wall was incised with a monopolar electrode in order to visualize the mass (Figure 7a). Then, a freer was used to mobilize the mucosa and push it laterally, in order to visualize the cystic mass (Figure 7b). The mass was further dissected with the use of the monopolar electrode and surgical instruments. Due to the tumor’s site, a standard mouth gag was used to gain exposure instead of the operating laryngoscope, which allowed for more space in the operating field. The operation lasted 90 min. No postoperative complications were noted. A histopathological examination revealed a cyst.

### 3.6. Case 13

**Figure 8:**
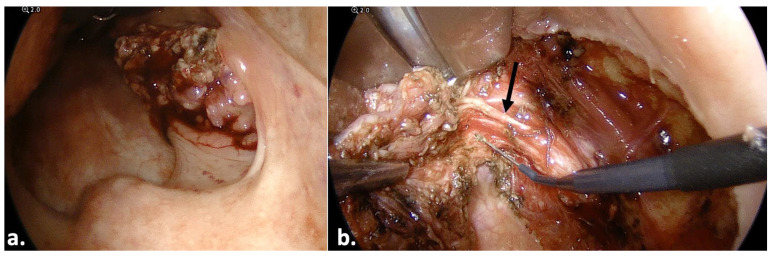
Transoral resection of a right oropharyngeal (tonsillar) tumor in a 56-year-old woman. Black arrow: the glossopharyngeal nerve.

A 56-year-old woman presented with dysphagia and unilateral pharyngeal pain in February 2021. An oropharyngeal examination revealed a tumor of the right tonsil. A computed tomography scan of the neck and thorax with contrast medium revealed no regional lymph node disease and no distant metastasis. A biopsy confirmed the diagnosis of an oropharyngeal squamous-cell carcinoma of the right tonsil. The patient underwent transoral pharyngeal endoscopic surgery in March 2021. All significant anatomical sites, e.g., the uvula, anterior and posterior palate arch and superior pharyngeal constrictor muscle, were visualized adequately (Figure 8a). The tumor was held medially with surgical forceps, which facilitated tumor resection with a monopolar electrode. Moreover, visualization with the endoscope allowed for identification of the glossopharyngeal nerve in the lower pole (Figure 8b). The operation lasted 35 min. We noted no bleeding. No further postoperative complications were noted.

## 4. Discussion

Pharyngeal surgery remains challenging, despite technological advancements that have led to the facilitation of tumor exposure, easiness of instruments’ handling, simplification of hemostasis and avoidance of open approaches. However, not all technological tools, i.e., robotic systems, microscopes and CO_2_ lasers, are globally available, due to their increased costs and/or limited surgical training. For this reason, we retrospectively investigated whether transoral pharyngeal surgery could be feasible with an endoscope-holding arm. Specifically, we intended to examine to what extent the endoscope-holding arm can offer an alternative to the microscope and robotic systems.

For transoral pharyngeal surgery, the standard in our university department is the microscope with a CO_2_ laser. Like every university department, we occasionally investigate new tools, like the endoscope-holding arm. Our retrospective search revealed 13 subjects with pharyngeal or posterior laryngeal tumors who were operated on with the endoscope-holding arm. The majority (11/13) were patients with oropharyngeal tumors. One patient presented with a tumor located in the hypopharynx (vallecula) and another one with a tumor located in the posterior larynx (arytenoid cartilage). Our study results implied that pharyngeal surgery is feasible with an endoscope-holding arm. However, there were certain challenges that are worth discussing.

Most oropharyngeal tumors were located in the lateral wall, including the tonsil (6/11) and the tongue base (4/11). The lateral wall tumors were exposed with the standard mouth gag used during tonsillectomy. Challenges in that area were the correct placement and angulation of the endoscope-holding arm. Surgeons should invest sufficient time into the placement of the endoscope according to their preferences. The correct position should allow for free movement of the surgeon’s hands.

On the contrary, the tongue-base tumors were exposed with an operating laryngoscope. The endoscope-holding arm and pair of instruments, i.e., a monopolar electrode and forceps, should have sufficient space inside the operating laryngoscope. Therefore, surgeons should use the largest possible laryngoscope. This is highly dependent on the patient’s anatomy. Thus, patients with a small mouth opening, large central and lateral incisors, macroglossia, mandibular prognathism, micrognathia, a history of previous radiotherapy, modified Mallampati score or high body mass index [24] might not be ideal candidates for tongue-base surgery with an endoscope-holding arm. The same applies to hypopharyngeal or laryngeal surgery. For these patients, only a small operating laryngoscope may be able to expose the tumor. Small operating laryngoscopes usually allow the passage of a maximum of two instruments. Therefore, the need for small operating laryngoscopes might require surgery with a microscope and CO_2_ laser, since the laser beam requires only a free direct line of sight and does not necessarily require space.

Our study results further implied that the endoscope-holding arm is a cost-efficient and safe oncological alternative to the microscope or even robotic systems. This can be explained by several observations made during the surgical treatment of the study subjects. First, the endoscope provided excellent visualization of significant structures, e.g., the glossotonsillar sulcus (Figure 4a), vessels (Figure 4b), glossopharyngeal sulcus (Figure 5a) and nerves (Figure 8b). The visualization of these structures highlighted its utility in the workup of cancer of unknown primary, where a tongue-base biopsy or resection is crucial [25] (Figure 6).

Second, the view with the endoscope can be angled, which is a significant advantage of the endoscope-holding arm over the microscope. Therefore, difficult-to-reach areas, which were encountered when using a microscope, were addressed using angled endoscopes.

Third, the endoscope-holding arm system allowed for bimanual surgical action. This was more easily applied to oropharyngeal surgery than to hypopharyngeal surgery, due to the larger space that was provided by the standard mouth gag. Limited space caused by small operating laryngoscopes was a significant disadvantage of the endoscope-holding arm.

Fourth, otorhinolaryngology departments often own neither a microscope nor robotic systems for the performance of pharyngeal surgery. Recently, there has been a tendency for the centralization of surgical care in large comprehensive cancer centers [26]. Due to the increased number of patients, these centers can invest in buying a microscope or robotic systems. The latter is not possible for otorhinolaryngology departments with fewer patients. However, these departments are able to borrow an endoscope-holding arm from the manufacturer for daily use and to carry out pharyngeal surgery. Moreover, if borrowing is not an option, the purchase price of an endoscope-holding arm is lower than that of the microscope or robotic systems, which are not globally available [27]. This may allow low- or even middle-income countries to perform pharyngeal surgery with a certain standard of quality.

Fifth, no training is required to use the endoscope-holding arm compared to the training required to use robotic systems [28]. Sixth, the endoscope-holding arm requires no setup in contrast to robotic systems.

This study further revealed that a monopolar electrode can offer an efficient alternative to the CO_2_ laser under certain circumstances. The use of a monopolar electrode was mainly feasible in oropharyngeal lateral or posterior wall tumors, where a standard mouth gag was used for exposure. The use of the monopolar electrode was more challenging through the operating laryngoscope, which was applied to oropharyngeal tongue-base or hypopharyngeal tumors. Similar to the relation between the endoscope-holding arm and the microscope or robotic systems, the monopolar electrode is cheaper than the CO_2_ laser. This implies that the monopolar electrode can be used instead of the CO_2_ laser in several places across the world, where the CO_2_ laser is not easily accessible.

Our results were in line with the study of Hintschich and coauthors [23]. The authors reported, among others, transoral treatment of five patients with head and neck tumors. Specifically, they described the case of a 68-year-old woman with a right dorsal hypopharyngeal wall. A laryngoscope was used for exposure, a 0° endoscope was used for visualization of the tumor with a 2 × 3 cm size, and a monopolar diathermy was used for resection. An R0 resection was performed. Based on that, the authors concluded that endoscopic surgery may not be limited to paranasal sinuses and the frontal skull base, but may expand to other operating sites, such as the pharynx and larynx. Furthermore, De Zoysa and coauthors reported on an endoscopic video-assisted transoral resection of lateral oropharyngeal tumors. The authors concluded that the technique facilitates oropharyngeal resection akin to transoral robotic surgery without the need for a robot [18].

The idea of an alternative to robotic systems or the microscope in transoral pharyngeal and laryngeal surgery is not new. Recent reports describe the facilitation of transoral pharyngeal and laryngeal surgery with an endoscope [29,30,31]. However, the endoscope-holding arm has significant advantages over the endoscope held by a person. For instance, the arm avoids shaky images due to fatigue, or a blurred field of vision due to the smudging of blood on the endoscope lens. Most importantly, it does not require an additional co-surgeon to guide the endoscope, which saves personnel resources.

Now that the study’s aims have been described, i.e., documentation of the advantages and disadvantages of the endoscope-holding arm, the limitations of the current study should be discussed. The main limitation of this case series is the absence of a comparison between groups. Instead, this case series serves more as a preliminary report. In our university department, certain outcome parameters may be systematically compared between patients who are operated on with the endoscope-holding arm/monopolar electrode and patients operated on with the microscope/CO_2_ laser. Outcome parameters could include the surgery duration, the level of the surgeon’s stress through measurement of heart-rate variability [32], tumor margins, hemoglobin before surgery and at the first postoperative day, pain using a visual analogue scale score and complications assessed using the Clavien–Dindo scale [33]. 

Furthermore, only six of the total thirteen cases were thoroughly described. The main reason for this was the availability of adequate photographic documentation in these six cases. Moreover, the presented cases should ideally cover all areas of the oropharynx, and we were able to do so with those for which we had documentation. In this instance, five of the six presented cases represented all parts of the oropharynx, i.e., the tonsil, tongue base and posterior pharyngeal wall, with the sixth case (Case 1) representing laryngeal tumors. The applicability of hypopharyngeal surgery, i.e., Case 11 with a hypopharyngeal tumor located in the vallecula, may be cross-checked with other cases with tumors located in the tongue base.

Lastly, in terms of limitations, the decision of which subject would undergo transoral pharyngeal surgery with the endoscope-holding arm was not based on a specific protocol. Instead, this decision was taken by each surgeon, based on the potential for an adequate oncological result. Therefore, selection bias cannot be excluded with certainty.

## 5. Conclusions

Transoral pharyngeal surgery is feasible with the endoscope-holding arm. Advantages over the microscope include an angled view. Advantages over robotic systems include the lack of training requirements, haptic feedback and a faster setup. Advantages over both the microscope and robotic systems include lower costs and easier availability. Visualization with the endoscope-holding arm is sufficient and may be similar to that of the microscope and the robot. Two disadvantages of the endoscope-holding arm are the need for proper repositioning during surgery and the reduced space that it causes when an operating laryngoscope is used for exposure of oropharyngeal tongue-base, laryngeal and hypopharyngeal tumors.

## Figures and Tables

**Table 1:** Characteristics of the patients who underwent pharyngeal surgery with the ENDOFIX.

Case	Age ^1^	Gender ^2^	Tumor Site	Location	T ^3^	Histology
1	65	M	Larynx	Arytenoid cartilage	2	Liposarcoma
2	53	W	Oropharynx	Tonsil	2	Squamous-cell carcinoma
3	70	W	Oropharynx	Tonsil	2	Squamous-cell carcinoma
4	51	M	Oropharynx	Tonsil	2	Squamous-cell carcinoma
5	50	W	Oropharynx	Lateral wall	2	Squamous-cell carcinoma
6	74	W	Oropharynx	Lateral wall	1	Squamous-cell carcinoma
7	65	W	Oropharynx	Tongue base	2	Squamous-cell carcinoma
8	72	W	Oropharynx	Tongue base	-	Normal (exclusion diagnosis)
9	45	M	Oropharynx	Tongue base	2	Squamous-cell carcinoma
10	45	W	Oropharynx	Tongue base	2	Adenoid cystic carcinoma
11	70	W	Hypopharynx	Vallecula	2	Adenoid carcinoma
12	65	W	Oropharynx	Posterior wall	-	Cyst
13	56	W	Oropharynx	Tonsil	2	Squamous-cell carcinoma

^1^ years; ^2^ W: woman; M: man; ^3^ tumor (clinical stage).

## Data Availability

The authors confirm that the data supporting the findings of this study are available within the article.

## References

[1] Steiner W. (1988). Experience in endoscopic laser surgery of malignant tumours of the upper aero-digestive tract. Adv. Otorhinolaryngol..

[2] Steiner W., Ambrosch P., Hess C.F., Kron M. (2001). Organ preservation by transoral laser microsurgery in piriform sinus carcinoma. Otolaryngol. Head Neck Surg..

[3] Steiner W., Fierek O., Ambrosch P., Hommerich C.P., Kron M. (2003). Transoral laser microsurgery for squamous cell carcinoma of the base of the tongue. Arch. Otolaryngol. Head Neck Surg..

[4] Laccourreye O., Villeneuve A., Rubin F., Holsinger F.C. (2019). Lateral pharyngotomy. Eur. Ann. Otorhinolaryngol. Head Neck Dis..

[5] Lim Y.C., Jeong H.M., Shin H.A., Choi E.C. (2011). Larynx-Preserving Partial Pharyngectomy via Lateral Pharyngotomy for the Treatment of Small (T(1~2)) Hypopharyngeal Squamous Cell Carcinoma. Clin. Exp. Otorhinolaryngol..

[6] Remmert C., Mansour N., Hofauer B., Scherer E.Q., Bas M., Bier H., Knopf A. (2017). Pharyngotomy in head and neck squamous cell carcinoma: Functional and oncological aspects. Acta Otolaryngol..

[7] Christopoulos E., Carrau R., Segas J., Johnson J.T., Myers E.N., Wagner R.L. (1992). Transmandibular approaches to the oral cavity and oropharynx. A functional assessment. Arch. Otolaryngol. Head Neck Surg..

[8] Michaelides P.L. (1996). Use of the operating microscope in dentistry. J. Calif. Dent. Assoc..

[9] Pecora G., Andreana S. (1993). Use of dental operating microscope in endodontic surgery. Oral Surg. Oral Med. Oral Pathol..

[10] Grillone G.A., Jalisi S. (2014). Robotic Surgery of the Head and Neck: A Comprehensive Guide.

[11] Scholfield D.W., Gujral D.M., Awad Z. (2020). Transoral Robotic Surgery for Oropharyngeal Squamous Cell Carcinoma: Improving Function While Maintaining Oncologic Outcome. Otolaryngol. Head Neck Surg..

[12] Schmitt N.C., Duvvuri U. (2015). Transoral robotic surgery for oropharyngeal squamous cell carcinoma. Curr. Opin. Otolaryngol. Head Neck Surg..

[13] Lallemant B., Moriniere S., Ceruse P., Lebalch M., Aubry K., Hans S., Dolivet G., Malard O., Bonduelle Q., Vergez S. (2017). Transoral robotic surgery for squamous cell carcinomas of the posterior pharyngeal wall. Eur. Arch. Otorhinolaryngol..

[14] Mahmoud O., Sung K., Civantos F.J., Thomas G.R., Samuels M.A. (2018). Transoral robotic surgery for oropharyngeal squamous cell carcinoma in the era of human papillomavirus. Head Neck.

[15] Tateya I., Muto M., Morita S., Miyamoto S., Hayashi T., Funakoshi M., Aoyama I., Higuchi H., Hirano S., Kitamura M. (2016). Endoscopic laryngo-pharyngeal surgery for superficial laryngo-pharyngeal cancer. Surg. Endosc..

[16] Kishimoto Y., Tateya I., Funakoshi M., Miyamoto S.I., Muto M., Omori K. (2020). Endoscopic laryngopharyngeal surgery for hypopharyngeal lesions. Oral Oncol..

[17] Watanabe A., Taniguchi M., Kimura Y., Hosokawa M., Ito S., Tsukamoto S., Sasaki S. (2017). Synopsis of transoral endoscopic laryngopharyngeal surgery for superficial pharyngeal cancers. Head Neck.

[18] Zoysa N., Sethi N., Jose J. (2017). Endoscopic video-assisted transoral resection of lateral oropharyngeal tumors. Head Neck.

[19] Mokhtari T., Abt N., Larson A., Holcomb A., Richmon J. Open Access Atlas of Otolaryngology, Head & Neck Operative Surgery. https://vula.uct.ac.za/access/content/group/ba5fb1bd-be95-48e5-81be-586fbaeba29d/Transoral%20Robotic%20Surgery%20_TORS_%20-%20Setup%20and%20Basics.pdf.

[20] Strauß G., Hofer M., Kehrt S., Grunert R., Korb W., Trantakis C., Winkler D., Meixensberger J., Bootz F., Dietz A. (2007). Ein Konzept für eine automatisierte Endoskopführung für die Nasennebenhöhlenchirurgie. HNO.

[21] Arnholt J.L., Mair E.A. (2002). A ‘third hand’ for endoscopic skull base surgery. Laryngoscope.

[22] Paraskevopoulos D., Roth J., Constantini S. (2016). Endoscope Holders in Cranial Neurosurgery: Part I-Technology, Trends, and Implications. World Neurosurg..

[23] Hintschich C.A., Fischer R., Seebauer C., Schebesch K.M., Bohr C., Kuhnel T. (2022). A third hand to the surgeon: The use of an endoscope holding arm in endonasal sinus surgery and well beyond. Eur. Arch. Otorhinolaryngol..

[24] Piazza C., Mangili S., Bon F.D., Paderno A., Grazioli P., Barbieri D., Perotti P., Garofolo S., Nicolai P., Peretti G. (2014). Preoperative clinical predictors of difficult laryngeal exposure for microlaryngoscopy: The Laryngoscore. Laryngoscope.

[25] Winter S.C., Ofo E., Meikle D., Silva P., Fraser L., O’Hara J., Kim D., Robinson M., Paleri V. (2017). Trans-oral robotic assisted tongue base mucosectomy for investigation of cancer of unknown primary in the head and neck region. The UK experience. Clin. Otolaryngol..

[26] Idrees J.J., Merath K., Gani F., Bagante F., Mehta R., Beal E., Cloyd J.M., Pawlik T.M. (2019). Trends in centralization of surgical care and compliance with National Cancer Center Network guidelines for resected cholangiocarcinoma. HPB.

[27] Mehta A., Cheng Ng J., Andrew Awuah W., Huang H., Kalmanovich J., Agrawal A., Abdul-Rahman T., Hasan M.M., Sikora V., Isik A. (2022). Embracing robotic surgery in low- and middle-income countries: Potential benefits, challenges, and scope in the future. Ann. Med. Surg..

[28] Sridhar A.N., Briggs T.P., Kelly J.D., Nathan S. (2017). Training in Robotic Surgery-an Overview. Curr. Urol. Rep..

[29] Chiesa-Estomba C.M., Larruscain-Sarasola E., Gonzalez-Garcia J.A., Sistiaga-Suarez J.A. (2023). Transoral endoscopic ultrasonic surgery (TOUSS) in head & neck unknown primary carcinoma investigation. Acta Otorrinolaringol. Esp. Engl. Ed..

[30] Fernandez-Fernandez M.M., Montes-Jovellar L., Parente Arias P.L., Ortega Del Alamo P. (2015). TransOral endoscopic UltraSonic Surgery (TOUSS): A preliminary report of a novel robotless alternative to TORS. Eur. Arch. Otorhinolaryngol..

[31] Sakthivel P., Ramanikanth T.V., Thakar A., Singh C.A., Sharma S.C. (2021). TransOral UltraSonic Surgery (TOUSS) assisted supraglottic laryngectomy: Expanding the spectrum of endoscopic head and neck surgeries. Oral Oncol..

[32] The A.F., Reijmerink I., van der Laan M., Cnossen F. (2020). Heart rate variability as a measure of mental stress in surgery: A systematic review. Int. Arch. Occup. Environ. Health.

[33] Bolliger M., Kroehnert J.A., Molineus F., Kandioler D., Schindl M., Riss P. (2018). Experiences with the standardized classification of surgical complications (Clavien-Dindo) in general surgery patients. Eur. Surg..

